# Human population movement can impede the elimination of soil-transmitted helminth transmission in regions with heterogeneity in mass drug administration coverage and transmission potential between villages: a metapopulation analysis

**DOI:** 10.1186/s13071-019-3612-7

**Published:** 2019-09-16

**Authors:** Carolin Vegvari, James E. Truscott, Klodeta Kura, Roy M. Anderson

**Affiliations:** 1 Department of Infectious Disease Epidemiology, Imperial College London, London Centre for Neglected Tropical Disease Research (LCNTDR), St Mary’s Campus, Praed Street, London, W2 1PG UK; 2 Department of Infectious Disease Epidemiology, School of Public Health, Faculty of Medicine, Imperial College London, St Mary’s Campus, Praed Street, London, W2 1PG UK; 3 The DeWorm3 Project, The Natural History Museum of London, London, SW7 5BD UK

**Keywords:** Soil-transmitted helminths, Elimination of transmission, Human population movement

## Abstract

**Background:**

Soil-transmitted helminth (STH) infections affect predominantly socio-economically disadvantaged populations in sub-Saharan Africa, East Asia and the Americas. Previous mathematical modelling studies have evaluated optimal intervention strategies to break STH transmission in clusters of villages. These studies assumed that villages are closed independent units with no movement of people in or out of communities. Here we examine how human population movement, for example, of seasonal migrant labourers, affect the outcome of mass drug administration (MDA) programmes.

**Results:**

We used a stochastic individual-based metapopulation model to analyse the impact of human population movement at varying rates on STH elimination efforts. Specifically, we looked at seasonal clumped movement events of infected individuals into a village. We showed that even if on average 75% of the entire resident population within a village are treated, an annual rate of 2–3% of the population arriving from an untreated source village can reduce the probability of STH elimination to less than 50% in high-prevalence settings. If a village is infection-free, an annual movement rate of 2–3% from an infected source village imposes a risk of re-introduction of STH of 75% or higher, unless the prevalence in the source village is less than 20%. Even a single arrival of 2–3% of the population can impose a risk of re-introducing STH of 50% or greater depending on the prevalence in the source village. The risk of re-introduction also depends on both the age group of moving individuals and STH species, since the pattern of cross-sectional age-prevalence and age-intensity profiles of infection in the human host are species-specific.

**Conclusions:**

Planning for STH elimination programmes should account for human mobility patterns in defined regions. We recommend that individuals arriving from areas with ongoing STH transmission should receive preventive chemotherapy for STHs. This can most easily be implemented if migration is seasonal and overlaps with treatment rounds, e.g. seasonal migrant labour. Moreover, transmission hotspots in or near treatment clusters should be eliminated, for example, by implementing appropriate water, sanitation and hygiene (WASH) measures and targeting treatment to individuals living in hotspots.

**Electronic supplementary material:**

The online version of this article (10.1186/s13071-019-3612-7) contains supplementary material, which is available to authorized users.

## Background

Soil-transmitted helminths (STHs) are a group of parasitic infections that affect 1.7 billion people worldwide [1]. STHs mainly occur in tropical and subtropical regions and predominantly affect socio-economically disadvantaged populations. The main STH species parasitizing humans are *Ascaris lumbricoides*, *Trichuris trichiura* and the two hookworm species *Ancylostoma duodenale* and *Necator americanus*. World Health Organization policy for STHs focuses on morbidity control in pre-school-aged and school-aged children (pre-SAC and SAC), aiming at reducing the prevalence of medium-to-heavy infections in pre-SAC and SAC to less than 1% by mass drug administration (MDA) and water, sanitation and hygiene (WASH) measures [2].

Field studies and cluster-randomised trials comprising regional clusters of villages, such as the TUMIKIA project and the DeWorm3 trial, have been set up to test if the WHO goals can be reached by MDA alone and if the interruption of transmission of STH by MDA is feasible [3, 4]. Interruption of transmission means that the parasite prevalence has become so low that sexual reproduction within hosts can no longer maintain the parasite population. The detailed mapping data generated by these types of projects illustrates a high level of spatial heterogeneity in both infection prevalence and intensity at multiple nested scales (clusters, villages, households) within the implementation unit. For example, epidemiological field observations after 25 years of MDA-based morbidity control programmes on Unguja Island, Zanzibar, found district-level prevalences of *A. lumbricoides*, *T. trichiura* and hookworm ranging from 0–16%, 9–45% and 2–13%, respectively [5]. At the village level, the variation was greater, ranging from 0% prevalence to more than 40% for *A. lumbricoides* and up to more than 60% for *T. trichiura*. Such heterogeneity may be due to a number of factors, either acting alone or in combination. These include intrinsic variation in the basic reproductive number, R_0_, due to social, demographic or environmental factors. MDA coverage and individual compliance patterns to drug uptake are undoubtably important in most endemic regions. MDA programmes typically report high levels of heterogeneity of treatment coverage within implementation units. For example, self-reported drug uptake in different villages within the same districts in Uganda ranged from 5–100% [6].

Spatial heterogeneity in disease prevalence in combination with human population movement have been recognised as important drivers for ongoing transmission in other infectious disease areas, for example, malaria, dengue, trachoma and HIV [7–10]. Observational evidence suggests that human mobility also plays a role in the transmission of neglected tropical diseases (NTDs) and that population displacement can be a reason for failure of NTD control programmes [11, 12]. For example, human African trypanosomiasis (HAT) has been introduced to southern Ghana by people moving away from environmental change and to Kinshasa, Democratic Republic of Congo, by people fleeing civil conflict [13, 14]. Smaller-scale but regular human mobility, for example, weekend tourism from urban to rural areas may expose hitherto healthy individuals to infectious reservoirs, and these individuals may import parasitic diseases to areas that were previously not endemic. This is how schistosomiasis was introduced to Belo Horizonte, Brazil, from surrounding rural areas [15].

Currently, there is little data on how human movement affects STH transmission and prevalence. However, given the heterogeneity in the distribution of STH infections, human movement patterns are likely to play a role in the transmission of STHs between different spatial units. The impact of infected individuals moving between locations on disease prevalence is likely to become more important as local populations move towards STH elimination of transmission, as MDA coverage rises year by year as reported by WHO [16]. This has been observed in the case of malaria control programmes. For example, in Sri Lanka which is close to eliminating malaria most cases are found in travellers returning from countries with endemic infection [17].

Stochastic simulation models of parasite transmission and control can be useful in making quantitative predictions on the impact of human mobility on STH prevalence. They can also help in exploring under what conditions human mobility is most important and what measure can be taken to mitigate its effect on STH control and elimination programmes. For the purpose of this study, we consider elimination as the extinction of STHs within a host population. However, human population movement probably also adversely affects the elimination of STHs as a public health problem. Moreover, individuals that are absent during MDA, but return later, decrease MDA coverage by non-compliance and hence the probability of success of MDA programmes [18]. For example, cross-border movement between Uganda and the Democratic Republic of Congo was one of the reasons for individuals being absent during the national MDA programme in Uganda, resulting in low coverage in the border region [6]. Thus, understanding human population movement and its impact on infection dynamics will be important for the successful conclusion of MDA programmes targeting STH elimination.

Human movement affecting STH transmission in villages can occur in two directions. First, individuals from an infection-free village or a village that receives treatment can move to another village where STHs are endemic and be exposed to the environmental reservoir of infectious material there (eggs or larvae in the soil). They become infected with a probability depending on their length of stay and return to their home village where they can deposit infectious material in the environment and thus transmit infection on to others. The second possibility is that individuals from a village where STHs are endemic arrive in an infection-free village or in a village that receives treatment. If the new arrivals are not treated, they will deposit infectious material in the environment that can infect the resident population. The second scenario should have a greater impact on re-introducing infection in a village, because individuals that long-term live in a location where STHs are endemic have more time to accumulate a greater worm burden. Therefore, in this paper we focused on the second scenario.

We investigated two questions. First, once elimination has been achieved in a village, what annual movement rate (in terms of individuals visiting from an infected source village as percentage of the local resident population per unit of time) re-establishes STH infection? Secondly, by how much does movement from an untreated source village decrease the probability of success of an ongoing MDA programme? We compared how different infection prevalences in the source village affect events in the village that receives incoming arrivals. In addition, we compared isolated, single movement events between villages *versus* regular, seasonal movement (for example, by seasonal migrant labourers) and movement of individuals of different age groups. We focused on two STHs here, *A. lumbricoides* and hookworm, because they have different age-prevalence and age intensity of infection profiles. The age-prevalence and intensity profiles of *A. lumbricoides* peaks in SAC, whereas for hookworm the prevalence and intensity of infection plateaus in adults.

## Methods

### Basic model description

The basic model is a stochastic simulation of the worm burdens of individual hosts within a population (for example, a village). The model has been described in detail elsewhere and has been used in previous simulation studies [19–24]. In brief, the model includes an age-structured host population in which the birth and death rates are representative of a typical low-income country. Individual hosts acquire STHs from an environmental reservoir of infectious material (eggs or larvae). The contact rates with the reservoir and the contribution to the reservoir are proportional and age-dependent. This leads to realistic parasite-specific prevalence-age and intensity-age profiles in the host population. In the model, parasites reproduce sexually within the host. This implies that below a critical infection prevalence the probability of both male and female worms being present within the same host becomes too small to sustain successful reproduction and hence transmission. This critical prevalence level is known as the transmission breakpoint [23]. The values of model parameters used in the simulations are given in Additional file 1: Table S1. Two model parameters play a major part in determining the equilibrium prevalence of infection in the host population; namely, the basic reproductive number (R_0_) which is a measure of the transmission intensity and the aggregation parameter k of the distribution of parasite numbers per host where k varies inversely with the degree of aggregation. The latter is the shape parameter of the negative binomial distribution that describes how “concentrated” or “aggregated” worm burden is among hosts. As the prevalence goes down, R_0_ decreases, while k increases. Different prevalences in individual village populations may be achieved by varying these two parameters. Table 1 lists the prevalence values used in our simulations together with the corresponding R_0_ and k values.

**Table 1:** STH prevalence values (any infection) in infected source villages used in simulations. The prevalence values are achieved by setting the basic reproductive number R_0_ and k (the shape parameter of the negative binomial distribution that describes the aggregation of parasites among hosts). The parameters required to achieve a desired prevalence value vary by species, because other parameters, for example, the worm life expectancy and transmission age structure, also differ by species

Prevalence		*A. lumbricoides*		Hookworm	
Level^a^	Value (%)	R_0_	k	R_0_	k
High	60–70	3.5	0.8	2	0.35
Medium	25–30	2.5	0.08	1.7	0.1
Low	< 20	2	0.04	1.6	0.05

^a^WHO risk categories based on prevalence for comparison [34]: high: > 50%; medium: 20–50%; low: < 20%

### Metapopulation model

To investigate the impact of defined annual rates of human population movement on the STH prevalence, we constructed a simple metapopulation model, consisting of two villages. We considered two basic scenarios. First, we simulated a pair of villages with 500 inhabitants each. One of the villages has eliminated STHs (no one is infected in this village), the other one has not eliminated STHs and does not receive treatment (infected individuals live in this village). We varied the prevalence of STHs in the infected or source village from low (< 20%), medium (25–30%) to high (60–70%).

We looked at two different types of human population movement: isolated, single events versus regular, seasonal movement. In isolated events a group of individuals from the village where STHs are endemic moves to the source village. In seasonal movement, a group of individuals from the source village moves to the infection-free village for half a year and back to their home village every year. Who moves is decided every year in the simulation. This means that the individuals who move to the infection-free village are not the same every year. This movement pattern is similar to seasonal migrant labour. For each movement type we varied the number of individuals from 0.1–10% of the local resident population in the infection-free village. During their stay in the infection-free village, individuals from the source village deposit infectious material (Table 2).

**Table 2:** Simulation scenarios. All scenarios were run assuming low (< 20%), medium (25*–*30%) and high (60*–*70%) STH prevalences in the source village. All scenarios were run for *A. lumbricoides* and hookworm. MDA scenarios were run with once- and twice-yearly community-wide treatment (75% coverage of all age groups except infants, random compliance) and with treatment of pre-SAC and SAC only (75% coverage, random compliance, treatment frequency according to WHO guidelines)

Frequency of travel	Re-introduction of STH by travellers after elimination	Travel during and after MDA
Single event	Main traveller group: young adults (15–35 years-old)	–
	Main traveller group: children < 15 years-old	–
Seasonal	Main traveller group: young adults (15–35 years-old)	Main traveller group: young adults (15–35 years-old) Treatment: community-wide, once a year
	–	Main traveller group: young adults (15–35 years-old) Treatment: community-wide, twice a year
	–	Main traveller group: young adults (15–35 years-old) Treatment: pre-SAC and SAC only, frequency following WHO guidelines
	Main traveller group: children < 15 years-old	–

We simulated the transmission dynamics for each scenario over a period of twenty years. As an outcome measure, we recorded in how many simulations out of 300 iterations, STH prevalence in the village that had previously eliminated reaches 50% of the equilibrium prevalence. We ran ten sets of 300 simulations for each scenario and plotted the mean and standard deviation of the measured outcomes. We chose the 50% threshold because populations in which the infection prevalence grows from 0% to more than 50% of the equilibrium value usually reach 100% of the equilibrium prevalence, but this may take many years. We examined if the age group of moving individuals affects the transmission between the two villages. We compare young adults (15–35 years-old) versus children (0–15 years-old). The latter would in reality usually accompany their mother.

To illustrate the difference between people from an infected source village moving to an infection-free village and people from an infection-free village temporarily moving to a village where STHs are endemic and returning to their home village, we simulates a scenario with seasonal movement of young adults or children for the latter case, too. In this simulation, individuals spend half a year in the source village and are exposed to the environmental reservoir there.

In the second basic scenario, we simulated a pair of identical villages, characterised by the same STH prevalence. One of the villages is part of a five-year MDA programme where individuals are treated once or twice per year with albendazole. We assumed 95% efficacy of the treatment [25], 75% coverage across all age groups and random compliance. The other village does not receive treatment. In an additional scenario, we assumed that only pre-SAC and SAC are treated with 75% coverage and random compliance and treatment frequency following the current WHO guidelines (which are undergoing revision in 2019) [1].

In the simulations with treatment, we only considered the impact of seasonal migrant labour (young adults from the source village travel to the treated village, but do not get treated themselves, and back to their home village each year) on the success of MDA in the treated village. We again ran the simulation for twenty years and vary the number of travellers and the prevalence in the two villages at baseline as above. As before, the group of individuals moving between villages is not the same every year. We also included a scenario with no travel between the two villages as a comparison on how likely the MDA programme would be to eliminate STHs in the absence of travel. As an outcome measure, we recorded the number of simulations out of 300 iterations in which the STH prevalence two years post cessation of MDA is below a threshold value that predicts with 95% probability whether the transmission breakpoint has been reached or not. As for the other scenarios, we ran ten sets of 300 simulations for each scenario and plotted the mean and standard deviation of the measured outcomes. The threshold value is 20% prevalence for *A. lumbricoides* and 9% for hookworm and has been determined in a previously published simulation study [24]. As human movement continues after the MDA programme ends, we record in how many simulations STHs have gone extinct 15 years after stopping MDA.

To examine if our results scale to larger population sizes, we repeated all analyses in which young adults from a source village where STHs are endemic move between two villages with a population size of 1000 people per village.

## Results

### Re-introduction of STHs after successful elimination

**Fig. 1:**
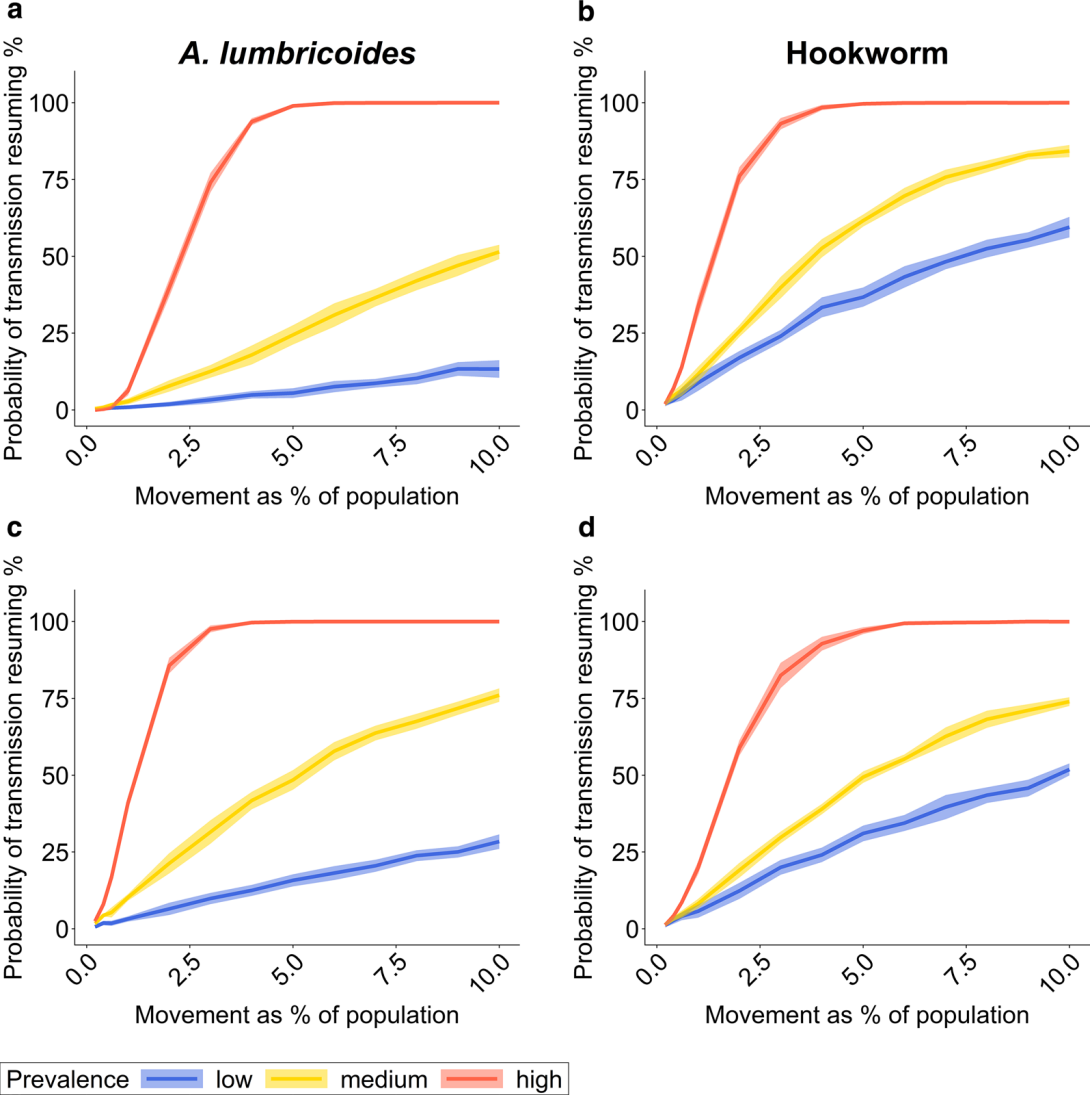
Probability of transmission resuming after an isolated movement event depending on the percentage of the population moving from a source village to an infection-free village and the STH prevalence in the source village. The probability estimate is based on the percentage of simulations out of 300 iterations in which the STH prevalence in the previously infection-free village reaches at least half of the equilibrium prevalence, given the transmission intensity and parasite aggregation in the source village. The solid line is the mean of ten sets of 300 iterative simulation runs. The shaded area is one standard deviation above and below the mean value. **a**, **b** Individuals moving from the source village are young adults (15*–*35 years). **c**, **d** Individuals moving from the source village are children (*< *15 years-old). **a**, **c** Results for *A. lumbricoides*. **b**, **d** Results for hookworm. Population size per village *n = *500. Prevalence levels: low: *< *20%; medium: 25*–*30%; high 60*–*70%

The risk of re-introducing STHs after successful elimination increases with both the number of individuals moving and the infection prevalence in the source village. In an isolated movement event from an infected source village, one or two individuals do not pose a significant risk of re-introducing STHs in the infection-free village, regardless of the prevalence in the source village. If the STH prevalence in the source village is high, however, as few as 10 individuals of either age group (2% of the population) pose a risk of re-introducing STHs to the infection-free village of more than 50%. If 3% of the population is moving from a high-prevalence source village, the risk of re-introduction can be 75% or more (Fig. 1). For medium or low STH prevalences in the source village, the risk of transmission resuming is markedly lower. But if 10% of the population from a low-prevalence area move to the infection-free village, the risk of transmission resuming can still be 50% or higher for hookworm. For *A. lumbricoides* the burden of disease is concentrated in SAC, and hence moving children pose a slightly higher risk of re-introduction than young adults (Fig. 1a, c). Conversely, in hookworm the infection prevalence is greater in adults. Consequently, the risk of re-introduction is higher for moving adults (Fig. 1b, d).

**Fig. 2:**
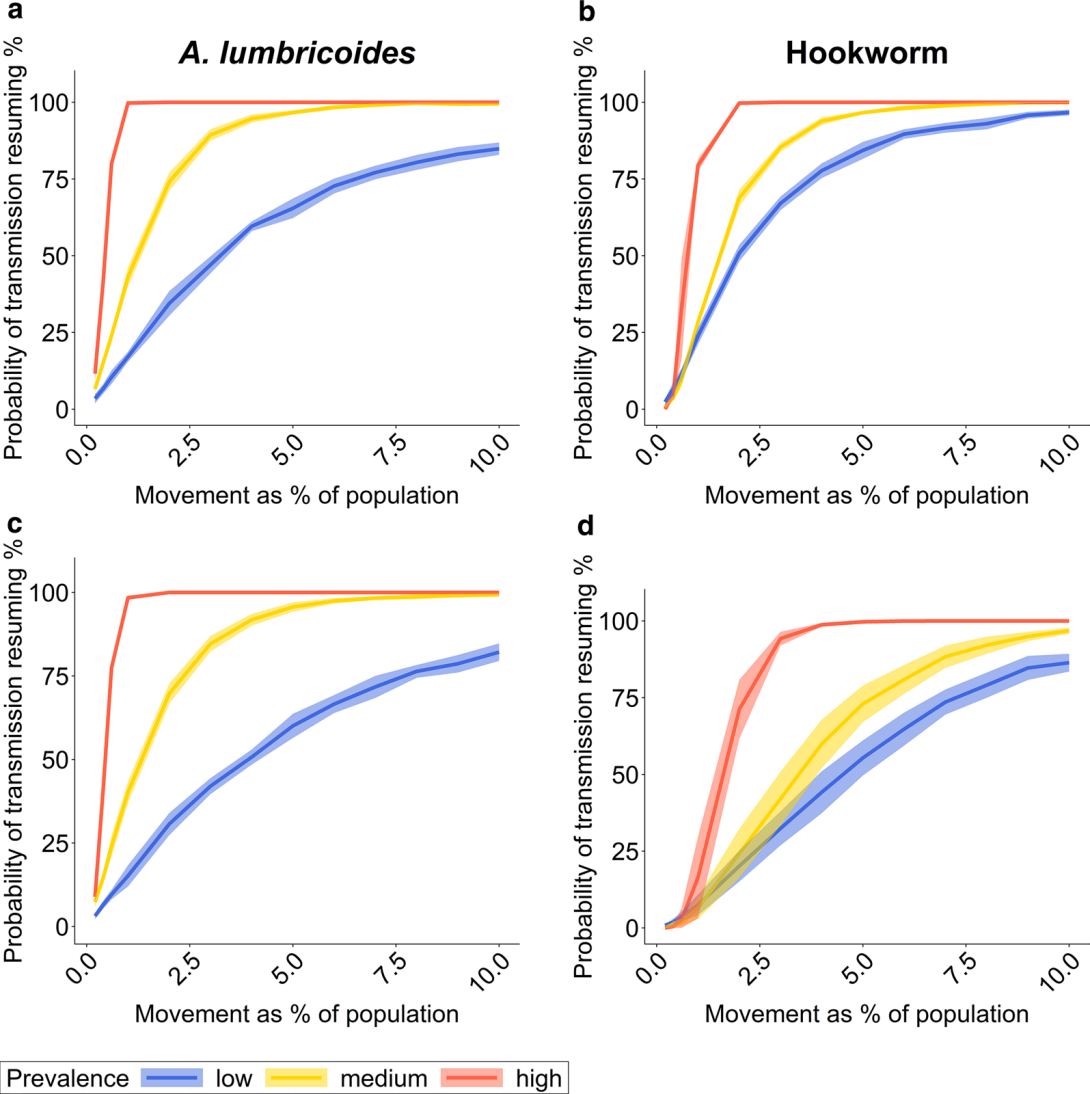
Probability of transmission resuming during twenty years of regular, seasonal movement. The probability of transmission resuming depends on the percentage of the population moving between villages and the STH prevalence in the source village. The probability estimate is based on the percentage of simulations out of 300 iterations in which the STH prevalence in the previously infection-free village reaches at least half of the equilibrium prevalence during the twenty-year observational period, given the transmission intensity and parasite aggregation in the source village. The solid line is the mean of ten sets of 300 iterative simulation runs. The shaded area is one standard deviation above and below the mean value. **a**, **b** Individuals moving between villages are young adults (15*–*35 years-old). **c**, **d** Individuals moving between villages are children (*< *15 years-old). **a**, **c** Results for *A. lumbricoides*. **b**, **d** Results for hookworm. Population size per village *n = *500. Prevalence levels: low: *< *20%; medium: 25–30%; high 60*–*70%

As expected, compared to single movement events, seasonal movement greatly increases the risk of re-introducing STH in a village that has previously eliminated STH transmission (Fig. 2). The rapid increase in the risk of STH re-introduction due to frequent, regular movement means that the impact of age group of the moving population becomes less important. In contrast, the prevalence of infection in the source village and the number of people moving between villages still have a pronounced effect. The differences in risk between *A. lumbricoides* and hookworm is not just related to the age profiles of infection. Differences in R_0_ and k (Table 1), and the parasite life expectancy are also important (Additional file 1: Table S1). For high-prevalence settings, the risk of re-introduction in relation to the number of people moving increases faster in *A. lumbricoides* because R_0_ is bigger in our simulations. For low-prevalence settings, the risk increases faster for hookworm if the individuals moving between villages are adult. The reason is that hookworm has a longer life expectancy than *A. lumbricoides* (two years *versus* one year). This has a stronger impact on transmission at low prevalences when stochastic (i.e. chance) transmission and death events become more important.

The scenario in which individuals from the infection-free village move to a source village where STHs are endemic and return to their home village poses a slightly lower risk for re-introducing infection (Additional file 2: Figure S1). At low prevalences, the difference between the two scenarios is less pronounced for hookworm. This can be explained again by the longer lifespan of hookworm compared to *A. lumbricoides*.

The simulated curves depicting the relationship between the percentage of a population moving between villages and the probability of transmission resuming in the infection-free village are not completely smooth. This is due to random effects in the stochastic simulations that are more pronounced at lower prevalence levels. The same patterns that we observe for village sizes of 500 people are observed if we assume village populations of 1000 people (Additional file 3: Figure S2). The magnitude of the effect of a given percentage of the population importing infection from a source village is the same for populations of either size, apart from stochastic fluctuations. This means that our conclusions are valid across a range of population sizes when simulations of movement are run as a percentage of the population.

### Human population movement during and after MDA implementation

**Fig. 3:**
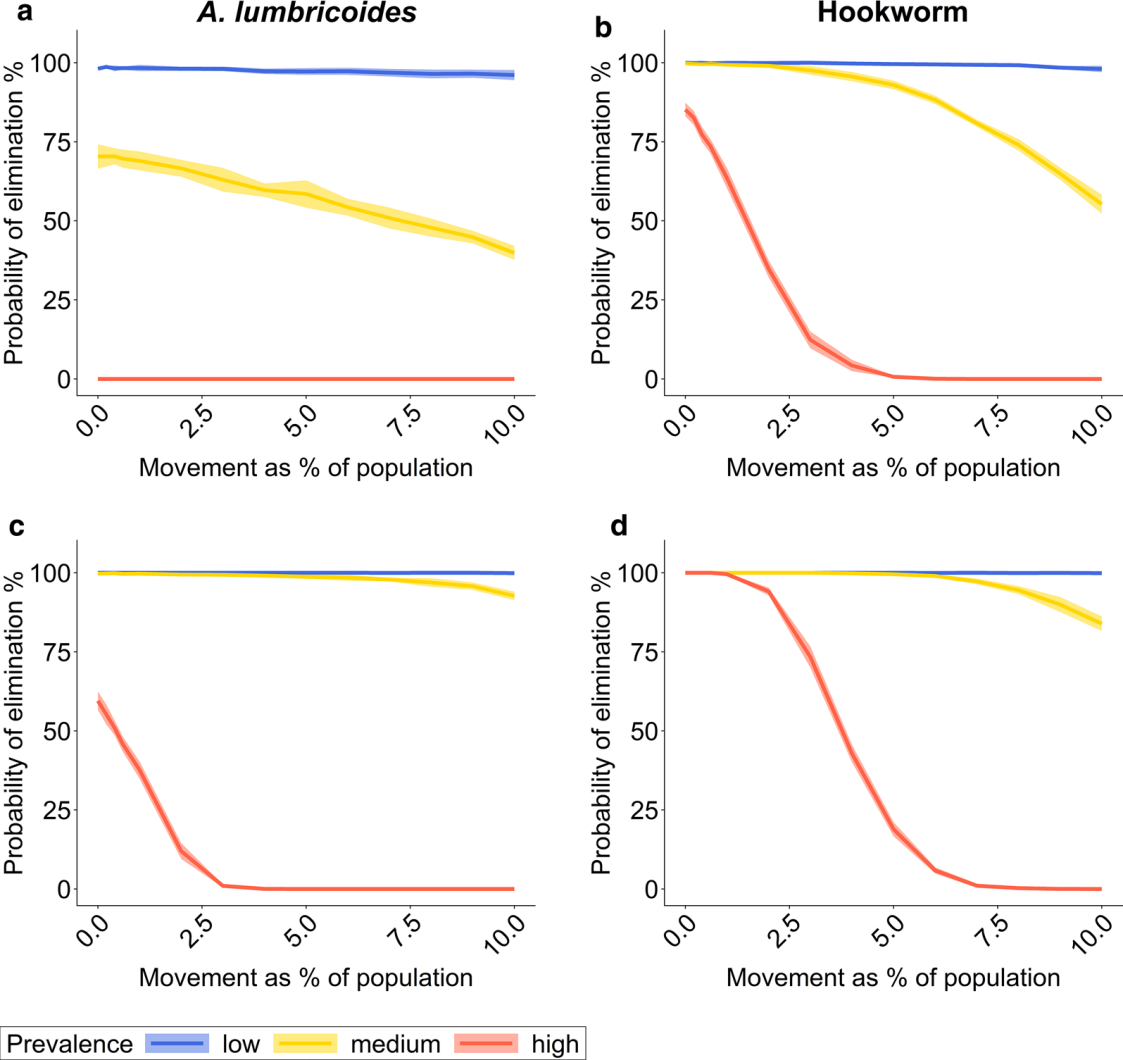
Probability of elimination determined two years after cessation of a five-year MDA programme dependent on annual movement rate during and after MDA. The probability of elimination depends on the percentage of the population moving between villages and the STH prevalence in the source village. The probability estimate is based on the percentage of simulations out of 300 iterations in which the STH prevalence in the treated village is beneath a previously determined threshold value that predicts with 95% probability whether the transmission breakpoint has been reached or not (20% for *A. lumbricoides*, 9% for hookworm). The solid line is the mean of ten sets of 300 iterative simulation runs. The shaded area is one standard deviation above and below the mean value. **a**, **b** The whole community receives MDA once a year with 75% coverage across all age groups. **c**, **d** The whole community receives MDA twice a year with 75% coverage across all age groups. **a**, **c** Results for *A. lumbricoides*. **b**, **d** Results for hookworm. Population size per village *n = *500. Prevalence levels: low: *< *20%; medium: 25*–*30%; high 60*–*70%

In the absence of human population movement, the probability of successfully eliminating STHs in a village by community-wide MDA is higher for lower baseline prevalences and lower transmission intensities (i.e. lower R_0_ values). Increasing treatment frequency raises the probability of MDA success if the baseline prevalence and the transmission intensity are high (Fig. 3). In our simulations, we used higher R_0_ values to achieve the desired baseline prevalences for *A. lumbricoides* compared to hookworm. Therefore, in our simulations the probability of elimination is higher for hookworm than *A. lumbricoides* in high-prevalence settings. Treating the whole community twice a year brings greater improvement for *A. lumbricoides* than for hookworm. This is in part a consequence of the longer life expectancy of hookworm by comparison with *A. lumbricoides*, since this parameter is an inverse determinant of bounce-back time to pre-treatment equilibrium; long-lived helminths bounce back to pre-control equilibrium population sizes slower than shorter-lived species [26, 27].

**Fig. 4:**
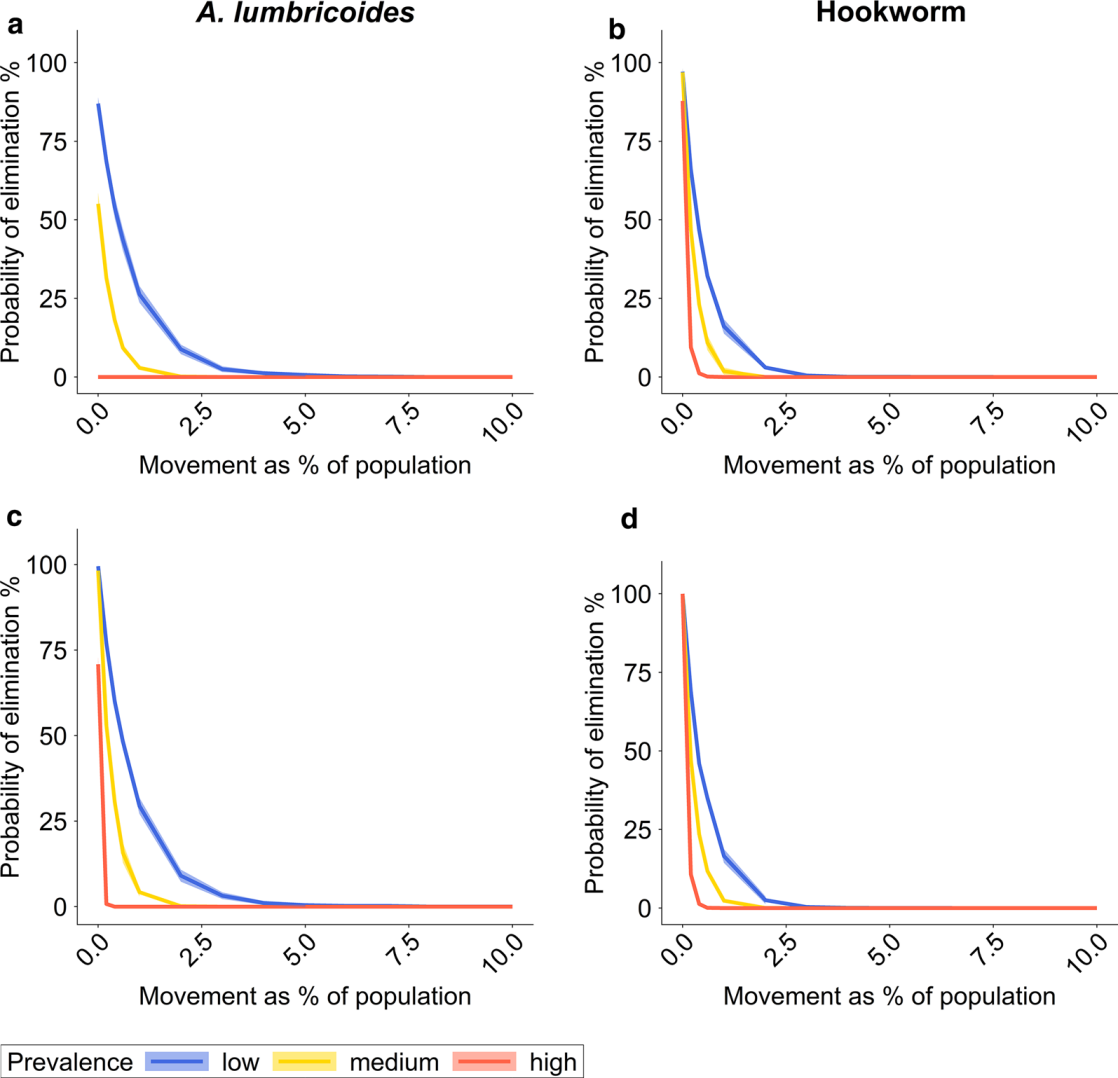
Probability of elimination determined fifteen years after cessation of a five-year MDA programme dependent on annual movement rate during and after MDA. The probability of elimination depends on the percentage of the population moving between villages and the STH prevalence in the source village. The probability estimate is based on the percentage of simulations out of 300 iterations in which STH infections have gone extinct in the treated village. The solid line is the mean of ten sets of 300 iterative simulation runs. The shaded area is one standard deviation above and below the mean value. **a**, **b** The whole community receives MDA once a year with 75% coverage across all age groups. **c**, **d** The whole community receives MDA twice a year with 75% coverage across all age groups. **a**, **c** Results for *A. lumbricoides*. **b**, **d** Results for hookworm. Population size per village *n = *500. Prevalence levels: low: *< *20%; medium: 25*–*30%; high 60*–*70%

At low prevalences seasonal movement has hardly any impact on MDA. At high prevalence levels for hookworm, the probability of elimination by MDA drops to 0, if only 5–7% of the population regularly spend time in an untreated source village. The same happens for high prevalence levels for *A. lumbricoides* if only 3% of the population move between villages (assuming treatment twice per year). If MDA is stopped after five years but human movement continues throughout the observation period, the probability of STH elimination assessed 15 years after MDA cessation falls below 50% if just 2% of the population are moving, even in low-prevalence settings for both *A. lumbricoides* and hookworm (Fig. 4).

If only pre-SAC and SAC are treated, as is frequently the case in practice, the probability of elimination is very low in the absence of human population movement (less than 20% for *A. lumbricoides* and less than 10% for hookworm) and diminishes further if humans move between locations. This is especially pertinent for hookworm as the main infection burden lies in adults (Additional file 4: Figure S3). If according to WHO guidelines, low-prevalence populations do not receive treatment, the probability of spontaneous extinction of STH is negligible (25% or less). It should be noted that in our simulations the prevalence in low-transmission settings is 10-20%. For prevalences below 10% spontaneous extinction in the absence of treatment may occur more frequently. As before, we observe the same results for larger population sizes of 1000 people per village (Additional file 5: Figure S4).

## Discussion

The results of our simulation analysis show that both regular and irregular human population movement can have a major adverse effect on the success of MDA-based STH elimination programmes, both during and after implementation. During implementation human movement between untreated and treated villages can reduce the probability of reaching the transmission breakpoint. Even if the breakpoint is reached during the MDA programme, or STH prevalence has reached 0%, ongoing inward movement from regions with endemic infection after MDA stops can re-introduce STHs into a population. Similar effects can be expected within an implementation unit, if coverage heterogeneity is high and some villages eliminate STH transmission, but others do not. It has been hypothesised before that transmission hotspots (small areas with high transmission intensity due to environmental, behavioural and socio-economic factors) can act as reservoirs from which re-introduction into disease-free and low-prevalence areas may occur [5].

The magnitude of the risk of re-introducing STHs into a population depends on the local context, such as the prevalence in the source village, the number of individuals moving between locations, how frequently they move and their age, and the dominant STH species. The metapopulation model used in this study was deliberately kept simple and generic to explore general principles of how different rates of human population movement and STH prevalences at the source of infection affect the outcome of STH elimination efforts by MDA. The model does not represent any particular geographical context, but it could be expanded to embrace a spatially structured individual-based stochastic model which is adapted to make more site-specific predictions and embed local culture and movement patterns.

We looked at four types of human movement patterns between two connected populations (single journeys of young adults or children, and seasonal return journeys of young adults or children). In reality metapopulations are usually more complex and human movement patterns are more diverse. For example, movements could occur on a daily basis, as in school visits, or be unidirectional and permanent, as in migration [7]. The impact of different types of human movement on STH prevalence is expected to be greater the more people move, the longer they stay in an area where STHs are endemic, and the more frequently people move between areas with different STH prevalences.

To make accurate predictions on the impact of human mobility on STH elimination and control programmes in specific geographical areas, reliable, high-quality infection prevalence and coverage data (preferably by village within an implementation unit) and data on human movement are required. Prevalence maps can be generated prior to the start of MDA programmes in a baseline evaluation. Coverage maps can be generated from coverage surveys as part of the monitoring and evaluation of MDA programmes. There are mainly two data sources on human movement, survey data and call data records (CDRs) from mobile phones. In addition, census data contains information on permanent relocations. While survey data asking participants targeted questions on their movement patterns can provide more detail, they are time-consuming to collect and may not always be reliable due to recall bias. CDRs are collected by mobile phone operators for entire populations but may not be accessible because of privacy concerns. Moreover, the spatial and temporal resolution of the data is limited by tower locations and individual calling behaviour [28]. Nevertheless, CDRs can be a valuable source of information on human movement patterns. For example, in a recent simulation study on schistosomiasis in the lower basin of the Senegal River, Senegal, CDRs in combination with hydrological data and data on the distribution of human settlements and snail populations have been used to explain spatial patterns of infection prevalence and intensity [29].

Phylogenetic analyses of biological samples that reveal ‘who infected whom’ may also provide information relevant to STH transmission dynamics. Early studies based on mitochondrial DNA markers in *N. americanus* found no correlation between geographical and genetic distance among surveyed sites in China. According to the authors, this may suggest uneven movement among the sites [30]. Another study, based on landscape genetics analyses, found that individuals from the same community in Nepal had acquired *A. lumbricoides* infection from different sources and that transmission foci were stable over time [31]. Following the sequencing of whole genomes of *A. lumbricoides* and *A. duodenale*, additional genetic markers may make it possible to trace transmission chains in more detail as is done for many viral and bacterial infectious agents [32]. Combining different data sources may allow us to estimate which human movement patterns are most important to STH transmission in a particular context. Stochastic simulation models can then help to estimate the impact of these movement patterns on MDA programmes targeting STH elimination.

Although MDA programmes may eliminate STHs in a closed population, re-infection can easily occur, especially after programme termination. Current estimates of the levels and frequencies of MDA coverage required to interrupt STH transmission are based on non-spatially structured transmission models. Although they have provided useful guidelines on the levels of coverage to be aimed for and which age groups of the population to be targeted, they do not account for heterogeneity in coverage between connected human communities.

Based on our simulation results, we recommend the following measures to avoid re-infection during or after MDA in settings with heterogeneous MDA coverage:

- Synchronise MDA with seasonal movement waves, where applicable (for example, agricultural seasonal migrant labour, or following major holidays when people may travel to visit family)
- Treat new arrivals/returners from outside the treated population (this would be particularly important in the case of population displacement) employing local health care workers with an intimate knowledge of the communities/villages and their populations
- Establish cross-border co-operations for the management of MDA programmes for STHs (as has been implemented, for example, with the E-8 initiative for malaria elimination programmes in Africa [17])
- Expand appropriate WASH measures to reduce the risk of re-infection after stopping MDA [33].

## Conclusions

Our simulation studies clearly indicate that strategic planning of STH elimination programmes should take human movement into account. Countries planning malaria elimination are advised by the WHO to estimate the risk of re-importing the disease using evidence-based methods as part of a feasibility analysis [7]. STH elimination programmes could benefit from a similar approach.

## Additional files

**Additional file 1: Table S1.** Model parameters used in simulations.

**Additional file 2: Figure S1.** Probability of transmission resuming after an isolated movement event depending on the percentage of the population moving from an infection-free village to a source village and back again and the STH prevalence in the source village. **a**, **c** *A. lumbricoides*. **b**, **d** Hookworm. Population size per village *n* = 500. Prevalence levels: low: < 20%; medium: 25–30%; high 60–70%.

**Additional file 3: Figure S2.** Probability of transmission resuming because of single or regular population movements, village size 1000 people. **a**, **b** A single, isolated movement event from an infected source village occurs in year 1 of the simulation. **c**, **d** Individuals from an infected source village move to the infection-free village and back to their home village every 6 months over the 20-year observational period. **a**, **c** *A. lumbricoides*. **b**, **d** Hookworm. Population size per village *n* = 1000. Prevalence levels: low: < 20%, medium: 25–30%, high 60–70%.

**Additional file 4: Figure S3.** Probability of elimination in the presence of seasonal human movement when only pre-SAC and SAC are treated. **a**, **c** *A. lumbricoides*. **b**, **d** Hookworm. Population size per village *n* = 500. Prevalence levels: low: < 20%, medium: 25–30%, high 60–70%.

**Additional file 5: Figure S4.** Probability of elimination determined two years or 15 years after cessation of a five-year MDA programme dependent on annual movement rate during and after MDA, village size 1000 people. **a**, **b**, **e**, **f** The whole community receives MDA once a year with 75% coverage across all age groups. **c**, **d**, **g**, **h** The whole community receives MDA twice a year with 75% coverage across all age groups. **a**, **c**, **e**, **g** *A. lumbricoides*. **b**, **d**, **f**, **h** Hookworm. Population size per village *n* = 1000. Prevalence levels: low: < 20%, medium: 25–30%, high 60–70%.

## Data Availability

Data used to inform the simulations are cited in Additional file 1: Table S1. R code to run the simulations can be downloaded from https://github.com/caro-veg/sth-hpm.
