# Supplementary material for: Human population movement can impede the elimination of soil-transmitted helminth transmission in regions with heterogeneity in mass drug administration coverage and transmission potential between villages: a metapopulation analysis
Source: Parasit Vectors. 2019 Sep 16;12:438. doi: 10.1186/s13071-019-3612-7 (PMC6745807; doi:10.1186/s13071-019-3612-7)
Supplement: Supplementary file 1 — Additional file 1: Table S1. Model parameters used in simulations. [file 13071_2019_3612_MOESM1_ESM.docx]

**Additional file 1: Table S1.** Model parameters used in simulations. The basic reproductive number R_0_ and the aggregation parameter k were varied to achieve pre-defined baseline prevalence values for both species. The values used are given in Table 1 in the main text. References for data from which parameter values were derived are listed below the table.

| Model parameter | *Ascaris lumbricoides* | Hookworm |
| --- | --- | --- |
| Population age structure, age-specific birth and death rates. | Demographic was data taken from 2003 Kenya Demographic and Health Surveys [1] | Demographic was data taken from 2003 Kenya Demographic and Health Surveys [1] |
| Relative age-dependent contribution to environmental reservoir  Parameters were fitted to epidemiological data  Age categories were chosen to fit the data | Values by age category:  1.6 (0-4 years), 1.54 (5-9), 1.0 (10-19), 0.9 (20-29), 0.6 (30-40) and 0.5 (45-70)  [2], [3] | Values by age category:  0.12 (ages 0-15), 1 (ages 15-25), and 0.07 (ages 25+)  [4], [3] |
| Relative age-dependent exposure to environmental reservoir. Assumed to be equal to relative age-dependent contribution to reservoir [5]  Parameters were fitted to epidemiological data  Age categories were chosen to fit the data | Values by age category:  1.6 (0-4 years), 1.54 (5-9), 1.0 (10-19), 0.9 (20-29), 0.6 (30-40) and 0.5 (45-70)  [2], [3] | Values by age category:  0.12 (ages 0-15), 1 (ages 15-25), and 0.07 (ages 25+)  [4], [3] |
| Average worm life expectancy  Assuming exponential distribution of variation in worm lifespan | 1 year [6, 7, 8, 9] | 2 years [10] |
| Density-dependence of female worm fecundity  Assuming exponential saturation | 0.07 [3] | 0.02 [5] |
| Egg production of female worms | On average 320 eggs per female worm per 41.7 mg sample of faeces | On average 3 eggs per female worm per 41.7 mg sample of faeces [11] |
| Survival of infectious material in the environmental reservoir | 2 months [12] | 30 days [10] |
| Drug efficacy | 0.95 [13] | 0.95 [13] |

**References**

1. Central Bureau of Statistics CBSK, Ministry of Health MOHK, Macro ORC: Kenya Demographic and Health Survey 2003. Calverton, Maryland, USA: CBS, MOH, and ORC Macro; 2004.

2. Elkins DB, Haswell-Elkins M, Anderson RM. The epidemiology and control of intestinal helminths in the Pulicat Lake region of Southern India. I. Study design and pre- and post-treatment observations on Ascaris lumbricoides infection. Transactions of the Royal Society of Tropical Medicine and Hygiene. 1986;80 5:774-92.

3. Truscott JE, Turner HC, Farrell SH, Anderson RM. Soil-Transmitted Helminths: Mathematical Models of Transmission, the Impact of Mass Drug Administration and Transmission Elimination Criteria. Advances in parasitology. 2016;94:133-98; doi: 10.1016/bs.apar.2016.08.002.

4. Sarkar R, Rose A, Mohan VR, Ajjampur SSR, Veluswamy V, Srinivasan R, et al. Study design and baseline results of an open-label cluster randomized community-intervention trial to assess the effectiveness of a modified mass deworming program in reducing hookworm infection in a tribal population in southern India. Contemporary clinical trials communications. 2017;5:49-55; doi: 10.1016/j.conctc.2016.12.002.

5. Coffeng LE, Truscott JE, Farrell SH, Turner HC, Sarkar R, Kang G, et al. Comparison and validation of two mathematical models for the impact of mass drug administration on Ascaris lumbricoides and hookworm infection. Epidemics. 2017;18:38-47; doi: 10.1016/j.epidem.2017.02.001.

6. Bethony J, Brooker S, Albonico M, Geiger SM, Loukas A, Diemert D, et al. Soil-transmitted helminth infections: ascariasis, trichuriasis, and hookworm. Lancet (London, England). 2006;367 9521:1521-32; doi: 10.1016/s0140-6736(06)68653-4.

7. Anderson R, Truscott J, Hollingsworth TD. The coverage and frequency of mass drug administration required to eliminate persistent transmission of soil-transmitted helminths. Philosophical transactions of the Royal Society of London Series B, Biological sciences. 2014;369 1645:20130435; doi: 10.1098/rstb.2013.0435.

8. Truscott JE, Hollingsworth TD, Brooker SJ, Anderson RM. Can chemotherapy alone eliminate the transmission of soil transmitted helminths? Parasites & vectors. 2014;7:266; doi: 10.1186/1756-3305-7-266.

9. Croll NA, Anderson RM, Gyorkos TW, Ghadirian E. The population biology and control of Ascaris lumbricoides in a rural community in Iran. Transactions of the Royal Society of Tropical Medicine and Hygiene. 1982;76 2:187-97.

10. Anderson RM, May RM. Helminth infections of humans: mathematical models, population dynamics, and control. Advances in parasitology. 1985;24:1-101.

11. Anderson RM, Schad GA. Hookworm burdens and faecal egg counts: an analysis of the biological basis of variation. Transactions of the Royal Society of Tropical Medicine and Hygiene. 1985;79 6:812-25.

12. Anderson RM, May RM. Population dynamics of human helminth infections: control by chemotherapy. Nature. 1982;297 5867:557-63.

13. Levecke B, Montresor A, Albonico M, Ame SM, Behnke JM, Bethony JM, et al. Assessment of anthelmintic efficacy of mebendazole in school children in six countries where soil-transmitted helminths are endemic. PLoS neglected tropical diseases. 2014;8 10:e3204; doi: 10.1371/journal.pntd.0003204.
